# Editorial: Optimizing radiotherapy for cervical cancer efficacy toxicity and brachytherapy integration

**DOI:** 10.3389/fonc.2026.1872192

**Published:** 2026-07-17

**Authors:** Irina Vergalasova, Chunling Jiang, Tao Song, Dorothy Lombe

**Affiliations:** 1 Rutgers Cancer Institute, Department of Radiation Oncology, New Brunswick, NJ, United States; 2 Jiangxi Cancer Hospital, Nanchang, China; 3 Cancer Center, Department of Radiation Oncology, Zhejiang Provincial People’s Hospital, Hangzhou, China; 4 Cancer Screening, Treatment and Support Services MidCentral, Palmerston North, New Zealand

**Keywords:** brachytherapy integration, cervical cancer radiotherapy, hypofractionation, imaging and radiomics, toxicity prediction

Cervical cancer remains a global health challenge that disproportionately affects the most vulnerable populations, reflecting persistent inequities in access to vaccination, screening, diagnosis and treatment. Despite the success of established image guided radiotherapy paradigms, including concurrent chemoradiotherapy and brachytherapy, continued innovation is required to sustain and extend these gains. This Research Topic brings together 13 contributions that collectively explore the evolving landscape of cervical cancer radiotherapy, highlighting the complex interplay between fractionation, workflow, imaging, toxicity management and emerging technologies such as artificial intelligence.

In their piece, Mallum et al. address resource limitation challenge by reducing the number of fractions required. In the highly conformal era of volumetric arc therapy hypofractionation is a reasonable approach whilst acknowledging and respecting the radiobiological properties of tumor type and radiosensitivity (Bi et al.). A shortened course of radiotherapy in resource limited setting has important implications both for the institution and the patient. Many patients’ undergoing treatment for cervical cancer are geographically displaced from their support systems. Acute toxicities typically emerge at approximately two weeks from commencement of treatment and peak in weeks for to six. A shortened treatment course may allow completion of therapy before the most severe toxicity phase, reducing both physical and socioeconomic burden. This represents a pragmatic form of optimization.

Beyond fractionation, toxicity prediction and management were addressed by the submissions from Xue et al., Luo et al., Zhao et al. and Fan et al. These studies present evidence on integration of imaging and predictive modelling, which may help match patients with appropriate treatment regimen. Wu et al. explored the use of a recombinant human endostatin to increase efficacy of treatment whilst maintain quality of life.

Workflow innovation emerges as a critical determinant of treatment quality. MRI has been established as a gold standard for brachytherapy imaging. However, it will remain unreachable for most radiotherapy centers in Low- and Middle-Income Countries (LMICs) and in smaller regional centers in developed countries. Taking advantage of the improved image quality of cone beam CTs (CBCT) on modern radiotherapy machines the community may look in the direction of implementing the findings of Karius et al. They introduced a cone beam-based approach to optimize needle placement and demonstrated measurable improvements in both geometric accuracy and dosimetry outcomes.

Integration of artificial intelligence is now an expected part of practice to optimize resource utilization. The review from Shi et al. provides an exquisite update on the position of AI integration in high dose rate brachytherapy for cervical cancer.^9^ In close relation to big data is the ability to use modelling in development of diagnostic tools. (Chen et al.; Sun et al.) Lastly, Zhang et al. and Alshdaifat et al. interrogate tumor behavior, response and diagnostic modelling to guide treatment allocation.

We hope readers will find this Research Topic provides meaningful insight and stimulates further progress in the optimization of cervical cancer radiotherapy.

